# Assessing the feasibility of a web-based domestic violence intervention using chronic disease frameworks: reducing the burden of ‘treatment’ and promoting capacity for action in women abused by a partner

**DOI:** 10.1186/s12905-016-0352-0

**Published:** 2016-11-24

**Authors:** Laura Tarzia, Carl May, Kelsey Hegarty

**Affiliations:** 1 Department of General Practice, The University of Melbourne, 200 Berkeley Street, Carlton, 3053 VIC Australia; 2 Faculty of Health Sciences, University of Southampton Highfield, Southampton, SO17 1BJ UK

**Keywords:** Domestic violence, Web-based intervention, Theoretical frameworks, Burden of treatment, Women

## Abstract

**Background:**

Domestic violence shares many features with chronic disease, including ongoing physical and mental health problems and eroded self-efficacy. Given the challenges around help-seeking for women experiencing domestic violence, it is essential that they be given support to ‘self-manage’ their condition. The growing popularity of web-based applications for chronic disease self-management suggests that there may be opportunities to use them as an intervention strategy for women experiencing domestic violence, however, as yet, little is known about whether this might work in practice.

**Discussion:**

It is critical that interventions for domestic violence—whether web-based or otherwise—promote agency and capacity for action rather than adding to the ‘workload’ of already stressed and vulnerable women. Although randomised controlled trials are vital to determine the effectiveness of interventions, robust theoretical frameworks can complement them as a way of examining the feasibility of implementing an intervention in practice. To date, no such frameworks have been developed for the domestic violence context. Consequently, in this paper we propose that it may be useful to appraise interventions for domestic violence using frameworks developed to help understand the barriers and facilitators around self-management of chronic conditions. Using a case study of an online healthy relationship tool and safety decision aid developed in Australia (I-DECIDE), this paper adapts and applies two theories: Burden of Treatment Theory and Normalisation Process Theory, to assess whether the intervention might increase women’s agency and capacity for action. In doing this, it proposes a new theoretical model with which the practical application of domestic violence interventions could be appraised in conjunction with other evaluation frameworks.

**Summary:**

This paper argues that theoretical frameworks for chronic disease are appropriate to assess the feasibility of implementing interventions for domestic violence in practice. The use of the modified Burden of Treatment/Normalisation Process Theory framework developed in this paper strengthens the case for I-DECIDE and other web-based applications as a way of supporting women experiencing domestic violence.

## Background

Domestic violence (DV) is defined as any behaviour within an intimate relationship that causes physical, psychological or sexual harm to those in the relationship [1]. Behaviours include intermittent acts of physical aggression; ongoing psychological abuse such as intimidation, constant belittling and humiliating; forced intercourse and other forms of sexual coercion and pervasive controlling behaviours such as isolating a person from their family and friends, monitoring their movements, and restricting access to information or assistance. One in three women globally report physical or sexual abuse in a relationship, with about one third of children witnessing violence at home [2]. In the US alone, 13.4% of women have been injured as a result of DV that included sexual violence, physical violence, or stalking by an intimate partner in their lifetime [3]. In Australia, the statistics are similar, with one in five women reporting physical or sexual abuse at the hands of a partner [4]. Women represent the majority of victims of domestic assaults and homicides by partners [5], however it is often the unrelenting ongoing emotional abuse that women say affects them the most. DV predisposes women and children to ill health and reduces their wellbeing, limiting their ability to take action [6, 7]. The cycle of abuse is strong. Left unrecognised, DV related trauma is cumulative in its impact and is associated with social, behavioural, emotional and cognitive problems in children, which persist into adulthood [6, 8].

The World Health Organisation defines chronic conditions as having complex causes, multiple risk factors, long periods without a diagnosis, prolonged course of illness and effects on functional ability [9]. DV has all these features. In Australia, it is the leading contributor to death, disability and illness for women of child bearing age [10]. Abused women use health services more frequently because of increased rates of emotional health issues (depression, anxiety, suicide, somatisation, post-traumatic stress disorder, substance abuse [11]) and physical health issues (chronic somatic complaints, reproductive problems and injuries [12]). There is evidence that women in abusive relationships often have associated conditions such as lower back pain, memory loss, diabetes, asthma, arthritis and digestive disease [13]. Women with a history of DV are also more likely to display negative health behaviours that present further health risks such as substance abuse, alcoholism, risky sex related behaviours (unprotected sex, early onset of sex), unhealthy diet related behaviours (bingeing, anorexia), when compared to women without a history of DV [14]. Studies show that the more severe the violence, the stronger its relationship to negative health outcomes for victims [15–21]. However, the magnitude of these health consequences contrasts starkly with its virtual invisibility within health practice, despite it being as common as asthma or diabetes. Up to five abused women per week per doctor attend clinics where this underlying condition is often not identified [22], and women typically make 7–8 visits to health professionals before disclosure [23].

In light of the contextual similarities between chronic disease and DV, there may be potential for some methods of intervention delivery that are effective for chronic disease self-management to also be useful in responding to abused women. Web-based and technological applications, for instance, are being increasingly utilised within a healthcare context, particularly for supported self-management of chronic conditions [24, 25]. In countries like Australia, the US and the UK, where over 70–80% of people are internet users [26–28], either via a desktop or via a portable device such as a phone or tablet, the potential market for these interventions is large. Greenhalgh and colleagues have suggested that the impetus behind the technological push at the policy level is that it will “generate social change” (p.2) and solve the problem of an overburdened workforce while also saving money [29]. A systematic review by Murray and colleagues looking at web-based applications in health suggested that there is potential for positive outcomes [30]. At the same time, however, questions are rightly being asked about whether web-based applications inappropriately burden patients, their families, and their social networks by encumbering them with the responsibility for decision-making, treatment and management of chronic conditions [31, 32]. It is therefore critical that web-based health applications be carefully designed so that they mobilise and facilitate capacity for action rather than adding to the workload of vulnerable patients. This is even more pertinent when the end users of applications are women experiencing DV who are already likely to be experiencing anxiety, depression, and stress [33].

## Discussion

The idea that web-based applications might be able to help women experiencing DV is still relatively new [34]. Face-to-face interactions with specialised DV services, counsellors or health care professionals may seem to be the obvious best response, considering that women say they value supportive listening, non-judgemental support and compassion [35]. However, recent studies have shown that there are many barriers to disclosure [36, 37] and women may go unsupported because they are unable or unwilling to seek help in a face-to-face setting. Providing appropriate security measures are in place (e.g. emergency exit buttons), the web offers anonymity and a forum where women can seek help without judgement. Pilot work in the United States [34] indicates that an online safety planning aid assisted women experiencing DV to reduce their decisional conflict and feel more supported. This concept is also being tested in New Zealand [38] and in Canada (NCT02258841). A study by Robinson-Whelan et al. [39], also in the US, found that a computer-based assessment tool for women with disabilities that utilised survivor vignettes, affirming messages, identification of warning signs, and the opportunity to self-report had a significant effect on abuse awareness. Young women who participated in a qualitative study by Lindsay et al. [40] were positive about the potential of a smartphone ‘app’ to provide personalised information about abuse in dating relationships, and to provide resources privately, safely, and non-judgementally.

### I-DECIDE: a case study from Australia

In Australia, an online healthy relationship tool and safety decision aid for women experiencing DV has been developed, called I-DECIDE. While I-DECIDE builds on the work being done in the US [34, 41], New Zealand [38], and Canada (NCT02258841), it differs from these tools in that it includes therapeutic and self-reflective exercises around healthy relationships rather than focusing purely on safety decisions. Briefly, the I-DECIDE website’s elements include: tools to promote self-reflection on the health of a woman’s relationship, safety and danger assessment, a priority-setting exercise, counselling elements such as a motivational interviewing tool [42] and a non-directive problem solving tool [43], and, most importantly, a tailored, individualised plan for action and list of resources that is based on the woman’s particular situation. The website and its benefits are described in more detail elsewhere [44]. While I-DECIDE seeks to be relevant to women at all stages of awareness, from those tentatively contemplating the idea that they might be in an unhealthy relationship through to women in crisis, the overarching aim is to allow them to self-inform, self-reflect, and self-manage in a secure, private space. I-DECIDE’s outcomes are currently being evaluated by a randomised controlled trial, the protocol for which is reported elsewhere [45].

**Fig. 1:**
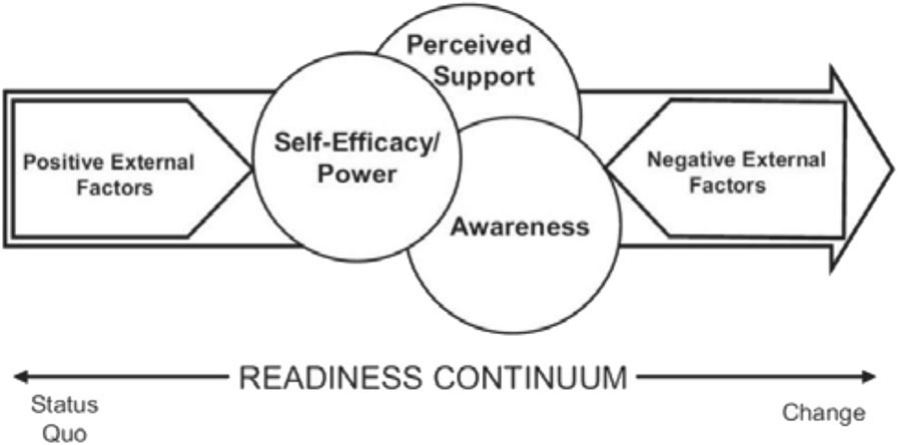
Psychosocial readiness model for IPV [46]

I-DECIDE is theoretically informed by the Psychosocial Readiness Model (PRM) [46]. The PRM takes into account the fluid and changeable nature of women’s journey towards positive action for safety and wellbeing. It focuses on three key internal factors: awareness, self-efficacy and perceived support, and suggests that interventions need to act on these elements in order to facilitate movement along the change continuum. At the same time, the model acknowledges the effects of external factors that are outside a woman’s control, and that these can either promote or hinder her level of readiness for change (see Fig. 1).

Whilst we have described elsewhere the conceptual development of I-DECIDE and why it ought to work in theory [44], the PRM is not an implementation model, and the use of additional theories may help to determine how it might work in practice. From an implementation point of view, I-DECIDE presents a special kind of challenge. Most implementation models assume motivation, freedom and capacity for action amongst the agents involved [47, 48]. Any constraints on agency are largely understood to be ‘internal’ problems of behaviour change or resistance. In the context of DV, however, as researchers have increasingly acknowledged [49], there are critical elements beyond the woman’s control that may have an impact on her ability to take action or make changes. These external factors can include structural inequities, the availability of resources (financial or social), the behaviour of the violent partner, or responses from the legal system. While the PRM acknowledges the role of these external factors in influencing women’s readiness for action, it does not examine how or why this occurs, or whether some factors are more relevant than others. To our knowledge, no other theories exist within the DV field that would enable this type of analysis. Most theoretical work in DV addresses the reasons why women choose to stay or leave a relationship, and most focus on individual factors rather than contextual ones [49].

In the absence of a specific DV-related implementation theory, and given the similarities between the experience of DV victimisation and chronic disease, we argue that by adapting two other theories, Burden of Treatment Theory (chronic disease) and Normalization Process Theory (implementation) [50, 51], we can develop a useful framework to assess the feasibility of implementing I-DECIDE. Although there are many other theories within the healthcare and chronic disease contexts, Burden of Treatment and Normalisation Process Theory together present a structural model that helps to understand variations in service utilisation and the importance of setting and context. Our aim here is not to provide empirical data or to test hypotheses regarding I-DECIDE—this will be done via the randomised controlled trial [45]. Rather, this paper proposes a theoretical framework that could be used to complement an RCT, and by which the feasibility of I-DECIDE’s uptake, use, and benefits to women in a real-world setting could be assessed.

### Women’s capacity for action in the context of DV

Burden of Treatment Theory explains the complex relationship between people, their support networks, and the healthcare system [51]. It highlights the *work* that people do in order to navigate and interact with health services, and outlines the key factors that can either contribute to, or alleviate, the burden of treatment experienced by a chronically ill patient. As we have argued earlier, there are clear parallels between the experience of chronic disease and the experience of DV, and therefore the idea that women in unhealthy or violent relationships may also experience a form of ‘burden of treatment’ when attempting to self-manage is not difficult to conceptualise. Abused women often suffer from anxiety, depression and complex trauma [52–55], are isolated from social networks and support systems [56], and have multi-morbidities due to both physical injuries and psychological stress [11, 12]. Their sense of self-efficacy is often undermined by being constantly put down and told that they are worthless by an abusive partner. Their opportunities for accessing care may be reduced as their behaviours may be carefully monitored [57]. Women may also be reluctant to engage with services that are branded as ‘domestic violence’ if they are not yet ready to acknowledge what they are experiencing as such [58].

**Fig. 2:**
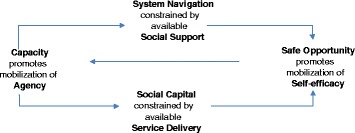
Attributes and resources

Although there are strong similarities between the chronic disease context and DV, there are also differences and particular nuances that need to be taken into account. Consequently, we have adapted the Burden of Treatment Theory to the particular context of DV, as shown in Fig. 2. Where the focus of Burden of Treatment is on the “capacity of individuals and their relational networks to interact with and utilise healthcare services” (p.3) [51], the focus of our model is on the capacity of women and their support networks to engage in strategies for safety and wellbeing.

Burden of Treatment Theory proposes that capacity and agency are resources that can be mobilised. When women are experiencing DV, their capacity to enact change is likely to be dependent on a variety of internal and external factors that, in turn, affect their level of agency in positive or negative ways. We define agency here as the things women might do to interact with others in the process of help-seeking for change. A woman is more likely to have agency if she receives good social support, whether it be by family members and friends, health practitioners, or other abused women, and this is acknowledged within the PRM and the broader literature on help-seeking in DV [59–63]. Considering that a woman needs to be able to engage in system navigation to mobilise the cooperation of others when experiencing DV (e.g. police, the legal system), and to negotiate the controls that are placed on her by these entities, social support is critical in helping her through this often-challenging process. Similarly, access to social capital in the form of financial or material resources (e.g. housing, bank account, employment), as well as social resources such as connections within the community, are critical to a woman’s journey towards positive change. At the same time, however, the ways in which service delivery is experienced by an abused woman can constrain her social capital. For instance, the ways in which community services brand themselves (e.g. ‘domestic violence’ versus ‘safe relationship’) and the language that is used in responding to women (e.g. ‘you should just leave’), can affect whether or not a woman feels able to draw on them for help. The degree to which other services such as utility companies, banks, and housing services are sensitive to the context of DV may also play a role in restricting or facilitating a woman’s access to social capital. The model also acknowledges the importance of having safe opportunities to access services or support networks. Many women experiencing DV are not able to achieve this, either because the abusive partner prevents them from seeking help, or because their location or physical circumstances make it difficult. Yet, having safe opportunities to access support is critical, as it is likely to promote and mobilise self-efficacy-another key component of the PRM. As shown in Fig. 2, we suggest that self-efficacy, or the extent to which a woman feels confident in being able to deal with adversity, directly influences her agency and capacity for action.

**Fig. 3:**
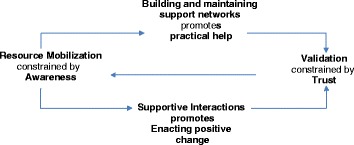
Attitudes and relations

In the context of this paper, it is important to also identify how the ‘work’ of self-managing DV might be enacted. Normalisation Process Theory (NPT) provides an evidence-based conceptual framework from which to assess the likelihood that an intervention or behaviour will become normalized or embedded into everyday practice [50]. It has been used in conjunction with Burden of Treatment Theory to understand the work that health professionals and patients do in managing chronic illness, and may also be usefully applied here. NPT’s key constructs focus on: sense-making or understanding the purpose of the work; clarity and cooperation around who is responsible for the work; the processes and mechanisms by which the work actually gets done; and retrospective follow-up and appraisal of the work. Again, we have adapted the constructs of NPT to the context of DV as shown in Fig. 3 below.

The concept of sense-making in the original NPT model very clearly corresponds to the key internal factor of *awareness* identified in the PRM. Awareness is vital for a woman and her support networks in order to understand the necessity of the actions being considered for positive change. A woman and the people supporting her are unlikely to take action if they do not understand that these actions are important. Research consistently supports the idea of “turning points” [64] as catalysts, where women’s awareness is suddenly raised by a threat to themselves or their children by the perpetrator and this prompts them to take action. As shown in Fig. 3, awareness constrains the mobilisation of resources (both material and social), as a woman is unlikely to access available supports if she is not aware that they are needed. Building and maintaining support networks is also critical to mobilising for positive change, as friends and family can play an important role in helping a woman actually carry out the actions she has chosen [65] (e.g. providing a safe place to stay if she wishes to leave the perpetrator) and can help her feel that she is not alone. Increasing the number of people in these support networks correspondingly increases the pool of skills and resources available within those networks, and promotes the provision of practical help that involves tasks that are realistic and achievable for the individual woman.

A woman and members of her support network then need to enact positive change, which is promoted through engagement and interactions (e.g. with service providers) that are affirming. This needs to be followed up with validation in the form of feedback that is encouraging and supports a woman’s choices (whatever those choices may be) [65], and increases her understanding that she is heading down a pathway to healing. Throughout this process, the establishment and maintenance of trust is critical, and directly influences the level of comfort a woman will have in her interactions with others.

### How does I-DECIDE mobilise capacity for action and help women enact positive change in the context of DV?

**Fig. 4:**
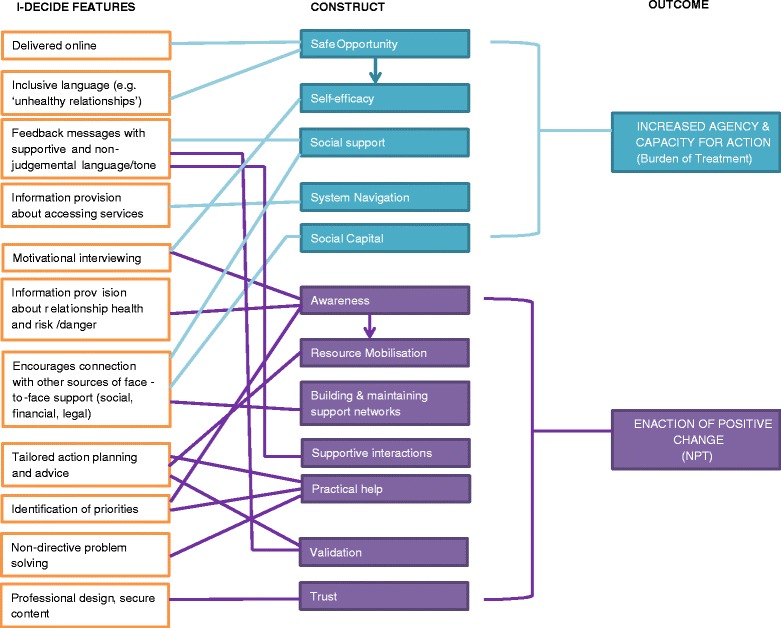
I‐DECIDE’s key elements and their relationship to BoT/NPT constructs

Assessing I-DECIDE through the lens of our adapted Burden of Treatment Theory and NPT framework allows us to hypothesise more rigorously about why it—and similar web-based applications—might work in practice. Abused women, like patients with chronic illnesses, experience eroded capacity for action that is especially pronounced given the constraints on their behaviour imposed by controlling partners. Web-based applications need to be able to counter this in order to be able to provide real benefits for women in practice. Figure 4 shows how key features and elements of I-DECIDE might act on particular constructs associated with agency and capacity to enact positive change and reduced burden of ‘treatment’. The model is based on a number of hypotheses, the accuracy of which will be tested through the ongoing randomised controlled trial [45].

I-DECIDE mobilises women’s agency and capacity for action in a number of ways. First, it acts directly to promote self-efficacy. It does this primarily by guiding women through motivational interviewing and non-directive problem solving activities that are designed to break down complex problems (e.g. whether her relationship is healthy, or what to do next) into manageable steps. As an online intervention, it also acts indirectly on the construct of self-efficacy by facilitating safe opportunities to seek help and support, potentially overcoming some of the inequalities in service delivery. Women with disabilities or whose mobility is restricted due to the perpetrator, as well as women who live rurally or remotely, can access valuable information and self-assessment about their relationship health and safety. The use of inclusive language such as ‘healthy relationships’ rather than ‘abuse’ or ‘domestic violence’ throughout the website, and particularly on the homepage, may also be helpful for women who do not immediately identify as a ‘victim’ [66], and may increase opportunities for help-seeking for these women as an alternative to face-to-face services.

In keeping with the theoretical framework of the PRM, I-DECIDE aims to increase a woman’s sense that she is supported, both by the website itself, and by encouraging her to speak with trusted friends, family members, or specialised services. According to the model, acting on social support in a positive way may increase a woman’s ability to navigate the system, particularly if she can recruit others to help her. I-DECIDE additionally acts directly to improve system navigation by providing tips and strategies for managing financial, legal, and safety issues, providing details about what to expect if a woman makes contact with services such as police, the courts, or shelters/refuges. It should be acknowledged that although I-DECIDE facilitates the acquisition of social capital in the form of social and financial resources, it cannot act on service delivery, and this may constrain the benefits that women are able to acquire from using the website. While it can prepare women for accessing services and the challenges they may face, I-DECIDE cannot influence the language that services might use in person, or any inappropriate responses that may eventuate. A future module for health professionals or services may be a useful addition to address this.

One of I-DECIDE’s primary goals is to raise awareness, particularly in women who may be unwilling or unable to self-identify as being abused. The healthy relationship module, which allows women to assess their relationship health, their level of fear, and level of safety in the relationship, is designed to promote critical self-reflection. The safety module, in which women complete the Danger Assessment [67] and the Composite Abuse Scale [68] to assess their level of risk for homicide, and level of abuse respectively, also encourages awareness. In particular, the interactive calendar element of the Danger Assessment, where women are asked to click days on which a violent episode occurred over the last year, may heighten awareness around the escalation of violence. Additionally, the interactive motivational interviewing tool encourages women to weigh up the pros and cons of the relationship with the abusive partner, with the hope that she will be able to make an informed choice about whether the relationship is meeting her needs. As Fig. 3 shows, awareness is not only important as an end-goal, as suggested in the PRM, but is also linked to the ability of women and their networks to mobilise resources. Although I-DECIDE acts directly on resource mobilisation through the tailored action plan, women are unlikely to be able to use resources effectively if they are unaware of when they might be needed.

Through encouraging women to connect with trusted friends, family members, other women who have experienced abuse, or service providers, I-DECIDE also has the potential to help women build and maintain supportive networks who can take on some of the ‘work’ involved in managing DV. This promotes the provision of practical help that extends beyond the online domain and into the real world setting. Furthermore, the culmination of I-DECIDE, where women are presented with a tailored ‘action plan’ of possible strategies, also represents practical help. The feasibility of the strategies suggested in a woman’s action plan are further enhanced through the non-directive problem solving module, which encourages step-by-step identification of possible options and solutions to carrying out the desired action.

Supportive interactions when engaging with the website are also helpful and encourage positive change. I-DECIDE does not tell a woman to ‘just leave’, but rather, is responsive to her plans for the relationship, whether she wishes to stay, leave, or has already left the abusive partner. The strategies suggested in the action plan are tailored to this decision, as well as to her individual priorities (e.g. financial stability, children’s safety, health and wellbeing). The website also provides validation through feedback and messaging that is supportive and non-judgemental, and this can assist women in clarifying their choices and actions. It has a focus on guiding women through a process of self-information, self-reflection and self-management, where they decide on what actions to take, rather than telling women what they should do. The team have consulted widely with women who have experienced abuse, as well as service providers, in order to determine the language, interface, and content, that would promote trust and a sense of legitimacy in the website. The secure login and password protection may also increase the level of trust, as women can be confident that a perpetrator will not view their responses.

## Conclusions

In examining I-DECIDE using Burden of Treatment Theory and, by extension, NPT, it is possible to assess the potential for I-DECIDE to mobilise capacity and enact positive change in women experiencing DV. As we have argued, DV shares many similarities with the experience of chronic disease, and theories around effective self-management can therefore provide a useful framework for assessing the feasibility of implementing interventions in a real-world setting. I-DECIDE follows accepted good practice for development of web-based interventions: (a) ensuring a strong theoretical underpinning [69, 70]; (b) basing them on face-to-face interventions known to be effective [71]; and (c) involving users in the development process [72]. We suggest that the modified Burden of Treatment/NPT framework developed in this paper strengthens the intervention further by supporting its real-world applicability. The framework could have broader applications in assessing future DV interventions in conjunction with randomised controlled trials. The theoretical insight it offers into *how* a DV intervention might mobilise capacity could provide benefits to researchers in the development phase to ensure that critical elements are targeted, or function as a means of process evaluation post-trial.

It is recommended that further work be done to explore the usefulness of the framework developed in this paper. While the use of chronic disease models to assess DV interventions makes sense, it is not clear to what extent they are actually relevant. DV is a complex and ‘wicked’ problem that is experienced differently by individual women, which makes it challenging to theorise about how interventions might work. Additionally, the original Burden of Treatment Theory and NPT on which our model is based are still undergoing refinement and development, which may result in a more nuanced understanding of self-management of chronic disease to build upon in future.
